# Gut microbial determinants of clinically important improvement in patients with rheumatoid arthritis

**DOI:** 10.1186/s13073-021-00957-0

**Published:** 2021-09-14

**Authors:** Vinod K. Gupta, Kevin Y. Cunningham, Benjamin Hur, Utpal Bakshi, Harvey Huang, Kenneth J. Warrington, Veena Taneja, Elena Myasoedova, John M. Davis, Jaeyun Sung

**Affiliations:** 1 Microbiome Program, Center for Individualized Medicine, Mayo Clinic, Rochester, MN USA; 2 Division of Surgery Research, Department of Surgery, Mayo Clinic, Rochester, MN USA; 3 Bioinformatics and Computational Biology Program, University of Minnesota, Minneapolis, MN USA; 4 Mayo Clinic Medical Scientist Training Program, Mayo Clinic, Rochester, MN USA; 5 Division of Rheumatology, Department of Medicine, Mayo Clinic, Rochester, MN USA; 6 Department of Immunology, Mayo Clinic, Rochester, MN USA; 7 Department of Health Sciences Research, Division of Epidemiology, Mayo Clinic, Rochester, MN USA

**Keywords:** Rheumatoid arthritis, Gut microbiome, Clinical disease activity index, Minimum clinically important improvement, Shotgun metagenomic sequencing, Machine-learning, Deep-learning neural network

## Abstract

**Background:**

Rapid advances in the past decade have shown that dysbiosis of the gut microbiome is a key hallmark of rheumatoid arthritis (RA). Yet, the relationship between the gut microbiome and clinical improvement in RA disease activity remains unclear. In this study, we explored the gut microbiome of patients with RA to identify features that are associated with, as well as predictive of, minimum clinically important improvement (MCII) in disease activity.

**Methods:**

We conducted a retrospective, observational cohort study on patients diagnosed with RA between 1988 and 2014. Whole metagenome shotgun sequencing was performed on 64 stool samples, which were collected from 32 patients with RA at two separate time-points approximately 6–12 months apart. The Clinical Disease Activity Index (CDAI) of each patient was measured at both time-points to assess achievement of MCII; depending on this clinical status, patients were distinguished into two groups: MCII+ (who achieved MCII; *n* = 12) and MCII− (who did not achieve MCII; *n* = 20). Multiple linear regression models were used to identify microbial taxa and biochemical pathways associated with MCII while controlling for potentially confounding factors. Lastly, a deep-learning neural network was trained upon gut microbiome, clinical, and demographic data at baseline to classify patients according to MCII status, thereby enabling the prediction of whether a patient will achieve MCII at follow-up.

**Results:**

We found age to be the largest determinant of the overall compositional variance in the gut microbiome (*R*^2^ = 7.7%, *P* = 0.001, PERMANOVA). Interestingly, the next factor identified to explain the most variance in the gut microbiome was MCII status (*R*^2^ = 3.8%, *P* = 0.005). Additionally, by looking at patients’ baseline gut microbiome profiles, we observed significantly different microbiome traits between patients who eventually showed MCII and those who did not. Taxonomic features include alpha- and beta-diversity measures, as well as several microbial taxa, such as *Coprococcus*, *Bilophila* sp. 4_1_30, and *Eubacterium* sp. 3_1_31. Notably, patients who achieved clinical improvement had higher alpha-diversity in their gut microbiomes at both baseline and follow-up visits. Functional profiling identified fifteen biochemical pathways, most of which were involved in the biosynthesis of L-arginine, L-methionine, and tetrahydrofolate, to be differentially abundant between the MCII patient groups. Moreover, MCII+ and MCII− groups showed significantly different fold-changes (from baseline to follow-up) in eight microbial taxa and in seven biochemical pathways. These results could suggest that, depending on the clinical course, gut microbiomes not only start at different ecological states, but also are on separate trajectories. Finally, the neural network proved to be highly effective in predicting which patients will achieve MCII (balanced accuracy = 90.0%, leave-one-out cross-validation), demonstrating potential clinical utility of gut microbiome profiles.

**Conclusions:**

Our findings confirm the presence of taxonomic and functional signatures of the gut microbiome associated with MCII in RA patients. Ultimately, modifying the gut microbiome to enhance clinical outcome may hold promise as a future treatment for RA.

**Supplementary Information:**

The online version contains supplementary material available at 10.1186/s13073-021-00957-0.

## Background

Rheumatoid arthritis (RA) is a chronic autoimmune inflammatory disease characterized by symmetric polyarticular inflammation and destruction primarily of the synovial joints, as well as of other organ systems [1]. The prognosis of RA has improved over recent decades in parallel with advancements in diagnosis and treatment, particularly the widespread use of biologic and targeted synthetic disease-modifying anti-rheumatic drugs (DMARDs) that enable many persons with RA to achieve low disease activity or clinical remission. However, the exact etiology and pathogenesis of RA are not yet fully understood [2]. In this regard, population-based studies have provided promising evidence that genetic factors contribute to RA onset [3–7]; however, the low concordance rate of RA in monozygotic twins largely suggests the role of non-genetic, environmental factors influencing the incidence of RA [4]. These non-genetic factors include smoking history [8], acute infections [9], and oral and gut microbiota [10].

During the past decade, the role of the gut microbiome in RA pathogenesis has been demonstrated by several experimental studies [11–16]. For example, Maeda et al. have shown increased sensitivity to arthritis (via auto-reactive T cell activation in the intestine) in germ-free SKG mice following fecal microbiota transplantation from early RA patients [15]. In addition, another study reported that inflammatory arthritis was strongly attenuated in K/BxN mice under germ-free (GF) conditions; however, the introduction of segmented filamentous bacteria restored splenic auto-antibodies, serum auto-antibodies, and T-helper 17 (Th17) cells [12]. Moreover, the role of gut microbiome in RA pathogenesis is further supported by the attenuation of arthritis in *Il1rn*^−/−^ mice by Tobramycin antibiotic treatment, which led to the decrease in relative abundances of gut commensals, such as *Helicobacter*, *Flexispira*, *Clostridium*, and *Dehalobacterium* [16].

Cross-sectional, human gut microbiome studies have elucidated the potential role of gut microbiome “dysbiosis” in RA [13, 14, 17, 18]. A study by Chen et al. found lower gut microbial diversity and species richness among RA patients compared to healthy controls; interestingly, patients using methotrexate (MTX) and hydroxychloroquine (HCQ) were observed to have higher gut microbiome diversity and richness than patients not on these medications, possibly indicating partial restoration of normal gut microbiome features with these treatments [13]. Additionally, patients with RA displayed significant improvement in disease activity after being provided with probiotics containing *Bacillus coagulans* [19] or *Lactobacillus casei* [20, 21], providing promising evidence towards probiotic therapies in RA treatment. Moreover, another study revealed significant associations between the relative abundance of gut microbial taxa (e.g., Euryarchaeota, Gammaproteobacteria, Erysipelotrichi, and Coriobacteriales) and the disease activity score on 28 joints (DAS28) [22]. Lastly, to demonstrate the potential of targeting the gut microbiome to modulate host immune response and to treat arthritis, Marietta *et al.* have shown that the oral administration of *Prevotella histicola*, which is a human gut-derived commensal bacterium, in transgenic mice expressing RA-associated DQ8 genes can suppress collagen-induced arthritis via regulation of the mucosal immune system [23].

Certainly, there has been a vast array of recent animal-model studies, cross-sectional case-control studies, and clinical trials showing that a perturbed gut microbiome is a key hallmark of RA. Yet, despite this wide range of novel findings, the association of the gut microbiome with minimum clinically important improvement (MCII) in disease activity in RA patients has yet to be closely examined. The MCII represents the minimal meaningful change (reduction) in quantitative disease activity, and is relevant to patients in terms of improvement in disease symptoms and associated clinical parameters [24]. Although the primary goal in RA management is to achieve and sustain clinical remission or, at least, low disease activity, the MCII in disease activity is also frequently used in clinical settings to evaluate the initial response to treatments. For this, there exists a variety of measurements to quantify RA disease activity, including the Disease Activity Score on 28-joints (DAS28), the Simplified Disease Activity Index (SDAI), and the Clinical Disease Activity Index (CDAI) [25, 26]. Among these quantitative indices, the CDAI is one of the most straightforward to use, as it is designed as a simple numerical addition of four components (clinician evaluator global assessment, patient global assessment, 28-swollen joint count, and 28-tender joint count), and does not require acute-phase reactant laboratory tests for its calculation [26].

As medicine evolves towards becoming a big data-centric and bioinformatics-driven discipline [27–29], one of the most promising translational opportunities with gut microbiome datasets arises from their predictive capabilities. In particular, through integrating key biological features (e.g., taxa, functions, genes) of the microbiome with cutting-edge, machine-learning approaches, large-scale data from gut microbiomes are positioned to inform various health and wellness applications and to guide or complement clinical practice. To this point, the gut microbiome has been demonstrated in recent years to facilitate detection of disease [30–34]; classification of disease subtypes and progression stages [35–37]; prediction of clinical outcomes and treatment efficacy [38–42]; personalized nutrition by prediction of postprandial glycemic response [43–45]; and estimation of chronological age [46]. Notably, in a recent study, by applying a random-forest machine-learning model to stool metagenomic data from treatment-naive, new-onset RA patients, Artacho et al. found that the gut microbiome can aid in the prediction of response to oral administration of methotrexate [47]. Taken together, these examples highlight the potential value of translating microbiome data into new prognostic tools for all areas of precision medicine.

In this study, by investigating the association of gut microbiome profiles from RA patients with MCII and with other patient factors, we demonstrate a computational approach for utilizing gut microbiome information to identify which patients are likely to show clinical improvement independent of baseline clinical features. To this end, we collect shotgun stool metagenomes from a pilot cohort of 32 patients with RA at two separate time-points (i.e., baseline and follow-up) approximately 6–12 months apart. First, we examine the association of gut microbiome with MCII in RA disease activity. Our results show that the status of whether clinical improvement is achieved (or not) is a significant factor contributing to the variance in gut microbiome taxonomic composition. Next, for each time-point, we examine microbiome properties (alpha- and beta-diversity, microbial taxa, and biochemical pathways) that differentiate patients who eventually show clinical improvement from those who do not. Afterwards, we identify taxonomic and functional features whose magnitude of and/or direction of change (from baseline to follow-up) varies differently between these two patient groups. Finally, we train a deep-learning neural network model on baseline microbiome, clinical, and demographic data to assess how well we can predict whether MCII in disease activity is attained. Encouragingly, we find that the neural network achieves a 90.0% balanced accuracy in leave-one-out cross-validation, with a compelling accuracy in those who showed clinical improvement (12 correctly predicted among 12 total). Overall, our study offers novel insights into how gut microbial signatures are connected to the trajectory of disease activity in RA, and provides proof-of-concept evidence that accurately forecasting MCII from a stool sample may be possible.

## Methods

### Patient enrollment, eligibility criteria, and sample collection

The study population consisted of consecutive patients with RA attending the outpatient practice of the Division of Rheumatology at Mayo Clinic in Rochester, Minnesota. Eligibility required patients to be adults 18 years of age or older with a clinical diagnosis of RA by a rheumatologist on the basis of the American College of Rheumatology/European League Against Rheumatism 2010 revised classification criteria for RA [48]. Patients were excluded if they did not comprehend English; were unable to provide written informed consent; or were members of a vulnerable population (e.g., incarcerated subjects). On the other hand, patients were eligible irrespective of use of any particular medication.

From 86 patients fulfilling the eligibility criteria, stool samples were collected from patients who had two outpatient visits approximately 6–12 months apart; whose clinical data (to assess CDAI and MCII) and demographic information were fully available at both clinical visits; who were not in clinical remission at both visits. In all, this study includes 32 participants, of whom 65.6% (21 of 32) were female.

For whole metagenome shotgun sequencing, stool samples were stored in our ongoing Mayo Clinic Rheumatology Biobank. This biorepository was created for long-term storage of diverse biological samples (e.g., serum, plasma, stool, white blood cells) from de-identified RA patients for use in research. Clinical and demographic data, including the numbers of tender and swollen joints, patient and evaluator global assessments, C-reactive protein (CRP, mg/L), smoking status, and titers for rheumatoid factor (RF, IU/mL) and anti-cyclic citrullinated peptide antibodies (ACPA), were collected from the electronic medical records. All patients provided written informed consent. The study was approved by the Mayo Clinic Institutional Review Board (no. 14-000616).

### Determination of minimum clinically important improvement (MCII) in RA disease activity

The CDAI of each patient was measured at two time-points. By taking into account the swollen joint count (of 28 joints), tender joint count (of 28 joints), and the global assessments of disease activity (scored 0–10 on a visual analog scale) by both patient and clinician, the CDAI is scored on a scale ranging from 0 to 76 points [25]. The level of disease activity can be interpreted as low (2.9 ≤ CDAI ≤ 10), moderate (10 < CDAI ≤ 22), or high (22 < CDAI), while CDAI ≤ 2.8 indicates the state of remission [49]. A decrease in CDAI of at least 1 for patients with low disease activity; of at least 6 for patients with moderate disease activity; and of at least 12 for patients with high disease activity between two consecutive visits is considered as MCII in RA disease activity [24]. Based upon these criteria, the study participants can be partitioned into two groups: (i) patients who showed clinical improvement (MCII+) and (ii) patients who did not show clinical improvement (MCII−) at follow-up visit.

### Stool sample collection, DNA extraction, and shotgun metagenome sequencing

Stool samples from patients with rheumatoid arthritis were stored in their house-hold freezers (−20 °C) prior to shipment on dry ice to the Medical Genome Facility Research Core at Mayo Clinic (Rochester, MN). Once received, the samples were stored at −80 °C until DNA extraction. DNA extraction from stool samples was conducted as follows: Aliquots were created from parent stool samples using a tissue punch, and the resulting child samples were then mixed with reagents from the Qiagen Power Fecal Kit. This included adding 60 uL of reagent C1 and the contents of a power bead tube (garnet beads and power bead solution). These were then vigorously vortexed to bring the sample punch into solution and centrifuged at 18,000× *g* for 15 min. From there, the samples were added into a mixture of magnetic beads using a JANUS liquid handler. The samples were then run through a Chemagic MSM1 according to the manufacturer’s protocol. After DNA extraction, paired-end libraries were prepared using 500 ng genomic DNA according to the manufacturer’s instructions for the NEBNext Ultra library prep kit (New England BioLabs). The concentration and size distribution of the completed libraries were determined using an Agilent Bioanalyzer DNA 1000 chip (Santa Clara, CA) and Qubit fluorometry (Invitrogen, Carlsbad, CA). Libraries were sequenced at 23–70 million reads per sample following Illumina’s standard protocol using the Illumina cBot and HiSeq 3000/4000 PE Cluster Kit. The flow cells were sequenced as 150 × 2 paired-end reads on an Illumina HiSeq 4000 using the HiSeq 3000/4000 sequencing kit and HiSeq Control Software HD 3.4.0.38. Base-calling was performed using Illumina’s RTA version 2.7.7.

### Quality filtration of sequenced reads

Sequenced reads were processed with the KneadData v0.5.1 quality-control pipeline (http://huttenhower.sph.harvard.edu/kneaddata), which uses Trimmomatic v0.36 [50] and Bowtie2 v2.3.2 [51] for removal of low-quality read bases and human reads, respectively. Trimmomatic v0.36 was run with parameters SLIDINGWINDOW:4:30, and Phred quality scores were thresholded at “< 30.” Illumina adapter sequences were removed, and trimmed non-human reads shorter than 60 bp in nucleotide length were discarded. Potential human contamination was filtered by removing reads that aligned to the human genome (reference genome hg19).

### Taxonomic and functional profiling of stool metagenomes

Taxonomic profiling was performed using the MetaPhlAn2 v2.7.8 [52] phylogenetic clade identification pipeline with default parameters. Briefly, MetaPhlAn2 classifies metagenomic reads to taxonomies based on a database (mpa_v20_m200) of clade-specific marker genes derived from ~ 17,000 microbial genomes (corresponding to ~ 13,500 bacterial and archaeal, ~ 3500 viral, and ~ 110 eukaryotic species). Microbes of viral origin and those that were labeled as either unclassified or unknown were excluded from further analyses. Afterwards, microbiome profiles were normalized using total sum-scaling (TSS) normalization to get the relative abundances (i.e., proportions) of microbial taxonomic ranks.

Functional profiling of annotated MetaCyc biochemical pathways of stool metagenomes was quantified using the HUMAnN v2.8 pipeline [53] with default parameters and with the UniRef90 EC-filtered database integrated into the pipeline. Similarly to the case with taxonomic ranks, MetaCyc pathways unmapped or unintegrated onto the UniRef90 EC-filtered database were discarded from further analyses, and relative abundances of the remaining MetaCyc pathways were calculated using TSS normalization.

### Permutational multivariate analysis of variance based upon taxonomic composition of microbial communities

Bray-Curtis distance matrices based on arcsine, square-root transformed relative abundances of microbial taxa (phylum to species) in stool metagenomes (collected at both clinical visits) were generated using the R “vegan” package v2.5.6. A permutational multivariate analysis of variance (PERMANOVA) [54] was performed on the distance matrix using the “adonis” function. *P* values for the test statistic (pseudo-F) were based on 999 permutations to assess the contribution of clinical and demographic characteristics (age group [age < 64 years; age ≥ 64 years], sex [male; female], smoking status [smoker; non-smoker], use of conventional synthetic disease-modifying anti-rheumatic drugs [csDMARDs], use of biologic disease-modifying anti-rheumatic drugs [bDMARDs], use of prednisone, and MCII patient group [MCII+; MCII−]) to the total variance in gut microbial community composition (of note, categorical age group was used due to the uneven and skewed distribution of continuous age). Intra-subject longitudinal variation was accounted for by constraining permutations to within visits using the “strata” argument. Both marginal (i.e., univariate analysis) and adjusted (i.e., multivariate analysis controlling for multiple covariates simultaneously) models were used to evaluate percent variance and significance of associations between gut microbiome composition and patient factors.

### Comparisons of alpha- and beta-diversity between MCII patient groups

Overall ecology of gut microbiomes was evaluated by calculating alpha-diversity (Fisher’s Index and richness) and beta-diversity (Bray-Curtis distance between all sample-pairs) based upon untransformed relative abundances of microbial species in each stool metagenome using the R “vegan” package v2.5.6. Multiple linear regression models (MLRMs) were then constructed using the R “stats” package v3.6.3 to determine the alpha-diversity indices that were significantly different between MCII+ and MCII− groups. MLRMs were adjusted for clinical and demographic characteristics that explained significant proportions of the variance in gut microbial community composition. Mann-Whitney *U* test was used to evaluate the statistical significance of the difference in beta-diversity between the patient groups.

### Identification of differentially abundant microbial taxa and biochemical pathways between MCII patient groups

To identify differentially abundant microbial taxa and biochemical pathways between MCII+ and MCII− groups (at either baseline or follow-up), MLRMs were constructed for arcsine, square-root transformed relative abundance of each taxon and pathway. All MLRMs were designed to model the relationship between a taxon/pathway and MCII patient group, while adjusting for clinical and demographic characteristics found to be significantly associated with gut microbiome compositional variance according to the aforementioned PERMANOVA analysis. Taxa and pathways were considered as differentially abundant between the two MCII patient groups if both of the following conditions were met: (i) the corresponding regression coefficient for the patient group was significant (*P* < 0.05) and (ii) detected in at least a third of all samples in order to avoid spurious associations based upon rarely seen events.

### Quantification of fold-change in gut microbial taxa and biochemical pathways

Microbial taxa and biochemical pathways detected in at least a third of all samples were considered for the calculation of fold-change (log_2_(FC)) from baseline to follow-up visit. As log_2_(FC) cannot be calculated if a taxon/pathway is absent (i.e., relative abundance = 0) at either of the visits, a small pseudo-count (1.0 × 10^−5^) was added to both the numerator and denominator when calculating fold-changes. Then, MLRMs were designed for each taxon and pathway to identify any significant differences (*P* < 0.05) in log_2_(FC) of relative abundances between the two MCII patient groups. All MLRMs were adjusted for clinical and demographic characteristics found to be significantly associated with gut microbiome compositional variance according to the aforementioned PERMANOVA analysis.

### Construction of neural networks for predicting MCII and CDAI

Two separate multi-layer (deep) feedforward artificial neural networks with stochastic gradient descent using back-propagation, which were provided by the Python version of the “H2O” package v3.26.0.3, were constructed to meet the following two objectives (i.e., output layer): (i) classify a patient as MCII+ or MCII− from all baseline gut microbiome (relative abundances of 176 taxonomic ranks and of 262 MetaCyc pathways), clinical (CDAI, use of medications [bDMARDs, csDMARDs, and prednisone], HAQ, pain, and CRP), and demographic data (age, sex, and smoking status). In other words, predict whether a patient will achieve MCII based upon all identifiable baseline features. This model’s predictive performance was evaluated by leave-one-out cross-validation on all baseline profiles. Furthermore, Gedeon’s technique [55], which uses a score function based upon hidden neuron activations, is implemented on the training set to calculate variable importance; and (ii) predict CDAI using the aforementioned microbiome, clinical (except for CDAI), and demographic data as input predictor variables for the neural network. Predictive performance of this second model was evaluated by a leave-one-patient-out cross-validation method. More specifically, in each cross-validation loop, both samples from the same patient were allocated as the internal validation set, while all remaining samples were used as the internal training set for constructing the neural network to predict CDAI scores of the allocated two samples. For both objectives, the default input parameters were used for model-training except for the following: Epochs = “10,000” and Random seed = “1234.” See http://docs.h2o.ai/h2o/latest-stable/h2o-docs/data-science/deep-learning.html for all parameters of the neural network and their default values. Data curation and model implementation was performed in Python v3.6.4 on individual cloud instances utilizing Amazon Web Services (AWS).

### MCII prediction with other machine-learning classifiers

Three different machine-learning models (logistic regression, random forest, and support-vector machines) from the “scikit-learn” package v0.24.1 were trained with the aforementioned baseline stool metagenome samples to create a classifier for predicting MCII status. The predictive performance of each classifier was evaluated by leave-one-out cross-validation. Of note, default options were used for the model training except for the following: logistic regression classifier, max_iter = “1000”; random forest, random_state = “1.”

## Results

### Study population

From a total of 86 patients with RA whose blood and/or stool samples were stored in our ongoing biobank, we identified 51 patients who had at least two available stool samples collected at least 6 to 12 months apart (102 total samples). From these 51 patients, we found 36 patients (72 samples) who had fully available clinical data and demographic information at both clinical visits, thereby leading to the exclusion of 15 patients (30 samples). We excluded an additional 4 patients (8 samples) from further analysis because they were in clinical remission at both clinical visits. Hence, this retrospective, observational cohort study includes 32 participants (64 samples), of whom 65.6% (21 of 32) were female.

**Fig. 1:**
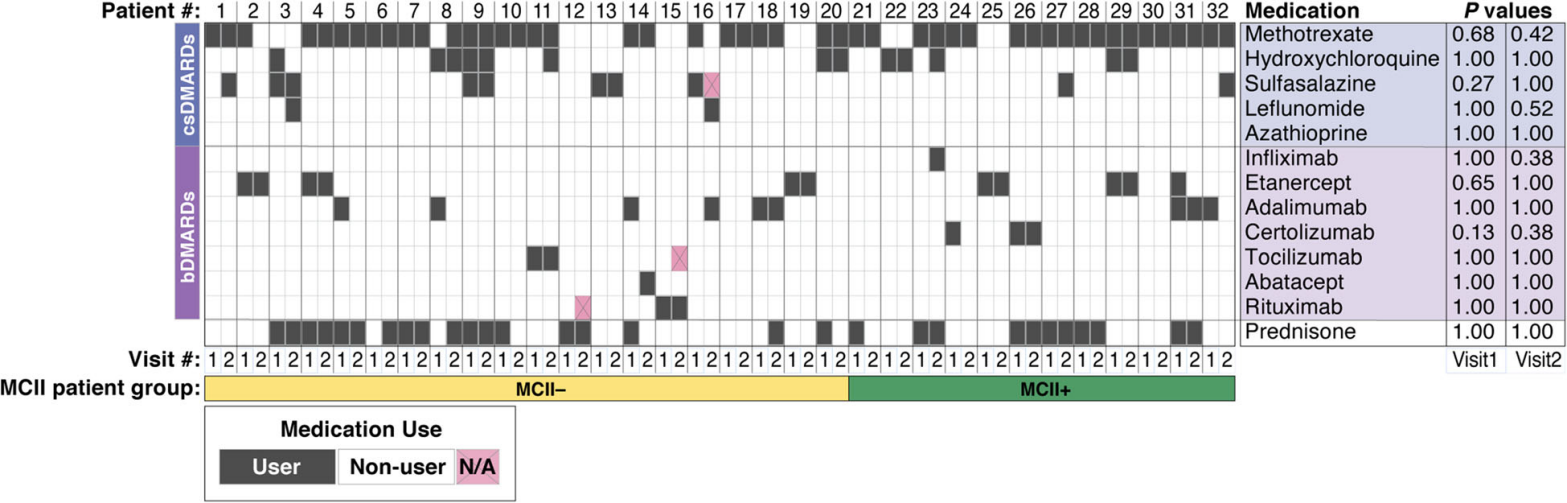
Overview of medication use by all 32 study participants shows no association with clinical improvement. *P* values from Fisher’s exact test (right) indicate statistical significance of association between MCII patient group and medication use/non-use at both visits. No significant association for any medication was found. bDMARDs, biologic disease-modifying anti-rheumatic drugs. csDMARDs, conventional synthetic disease-modifying anti-rheumatic drugs. MCII, minimum clinically important improvement. MCII+, patients who showed MCII. MCII−, patients who did not show MCII. N/A, not available

At the time of baseline stool sample collection, the patients had established disease with a mean age of 64.9 years (s.d. = 11.0), and a mean disease duration of 8.2 years (s.d. = 8.2). A summary of the patient enrollment, eligibility criteria, and sample collection protocol is provided in the “Methods” section. At baseline, all patients were on treatment with biologic disease-modifying anti-rheumatic drugs (bDMARDs, 46.9%), conventional synthetic disease-modifying anti-rheumatic drugs (csDMARDs, 87.5%), or prednisone (46.9%). For any medication, no association was found between its use (at either baseline or follow-up visit) and MCII in RA disease activity (Fig. 1), showing the critical need for more effective predictors of clinical improvement in RA. Baseline and follow-up visits were separated by a mean duration of 9.5 months (s.d. = 3.6 months), which was numerically longer for patients who attained MCII than for patients who did not attain MCII though not statistically significant (median 363 vs. 252 days, respectively; *P* = 0.08, Mann-Whitney *U* test). At all instances of stool sample collection, disease activity of patients varied from remission to high disease activity, with a mean CDAI of 16.3 (s.d. = 13.7) and 13.6 (s.d. = 11.6) at baseline and follow-up, respectively.

In total, 12 of the 32 (37.5%) total study participants achieved MCII in RA disease activity at their follow-up visit. The average change in CDAI for these 12 patients was –16.7 (s.d. = 12.8) units, which was, as expected, significantly different from the average change in CDAI of 5.7 (s.d. = 8.9) units for the remaining 20 of 32 (62.5%) patients who did not show improvement in RA disease activity (*P* = 6.9 × 10^–6^, Mann-Whitney *U* test). We used Fisher’s exact test to identify significant differences in categorical variables (e.g., age group, sex, smoking status, medication use, presence of rheumatoid factor or anti-cyclic citrullinated peptides antibodies), and Mann-Whitney *U* test to identify significant differences in continuous clinical measurements (CDAI, health assessment questionnaire [HAQ], swollen joint count [SJC], tender joint count [TJC], C-reactive protein [CRP], patient’s and physician’s health status assessment) between two patient groups: MCII+ (i.e., patients who showed MCII in disease activity based upon the change in CDAI from baseline to follow-up visit) and MCII– (i.e., patients who did not show MCII) (Table 1). At baseline, we found a significant association between MCII patient group (i.e., MCII+ and MCII–) and CDAI (*P* = 0.03). At follow-up visit, we found the following factors to be significantly associated with MCII patient group: CDAI (*P* = 1.9 × 10^–3^), change in CDAI from baseline (*P* = 6.9 × 10^–6^), pain (VAS) (*P* = 2.8 × 10^–3^), TJC (*P* = 0.01), patient global evaluation of disease activity (pt_vas) (*P* = 6.3 × 10^–3^), and provider global evaluation of disease activity (md_vas) (*P* = 0.01). No significant difference in age between the two patient groups was observed at either baseline or follow-up (*P* = 0.09).

**Table 1:** Demographic and clinical characteristics of the study population

Patient characteristics	Visit 1 (baseline)			Visit 2 (follow-up)		
	Minimum clinically important improvement patient group^*^		*P* value^**^	Minimum clinically important improvement patient group^*^		*P* value^**^
	MCII+ (*n* = 12)	MCII− (*n* = 20)		MCII+ (*n* = 12)	MCII− (*n* = 20)	
Duration between visits (days), median [Q1, Q3]^a^	-	-	-	363.0 [302.5, 377.5]	252.0 [212.0, 341.3]	0.08
Sex			0.70			0.70
Female, *n* (%)	7 (58.3)	14 (70.0)		7 (58.3)	14 (70.0)	
Male, *n* (%)	5 (41.7)	6 (30.0)		5 (41.7)	6 (30.0)	
Age, mean ± s.d.	68.0 ± 12.8	63.2 ± 9.7	0.09	68.9 ± 13.1	64.0 ± 9.7	0.09
< 64 years, *n* (%)	3 (25.0)	10 (50.0)	0.27	3 (25.0)	10 (50.0)	0.27
≥ 64 years, *n* (%)	9 (75.0)	10 (50.0)		9 (75.0)	10 (50.0)	
Smoking status			0.52			0.27
Smoker, *n* (%)	0 (0)	2 (10.0)		0 (0)	3 (15.0)	
Non-smoker, *n* (%)	12 (100)	18 (90.0)		12 (100)	17 (85.0)	
Clinical measurements						
^b^CDAI, median [Q1, Q3]	19.8 [12.6, 30.1]	9.3 [6.4, 13.7]	0.03	2.7 [1.0, 7.7]	14.0 [10.1, 21.7]	1.9 × 10^−3^
ΔCDAI, median [Q1, Q3]	-	-	-	− 15.1 [− 19.2, − 10.0]	2.7 [− 0.2, 9.8]	6.9 × 10^−6^
^c^HAQ, median [Q1, Q3]	0.6 [0.4, 0.9]	0.8 [0.3, 1.1]	0.57	0.4 [0.3, 0.8]	0.7 [0.4, 1.2]	0.09
Pain (VAS^d^ 0–100 mm), median [Q1, Q3]	41.0 [11.8, 67.8]	26.5 [14.8, 56.3]	0.61	9.5 [8.0, 36.0]	63.0 [31.8, 78.5]	2.8 × 10^−3^
28-swollen joint count (SJC), median [Q1, Q3]	6.5 [0.0, 13.3]	1.5 [0.0, 4.3]	0.12	0.0 [0.0, 2.0]	1.0 [0.8, 4.8]	0.08
28-tender joint count (TJC), median [Q1, Q3]	4.5 [0.0, 15.0]	1.5 [0.8, 5.3]	0.45	0.0 [0.0, 0.5]	3.0 [2.0, 9.0]	0.01
^e^pt_vas, median [Q1, Q3]	45.5 [28.8, 59.0]	28.0 [14.5, 52.3]	0.56	10.5 [8.0, 35.0]	49.0 [32.0, 71.3]	6.3 × 10^−3^
^f^md_vas, median [Q1, Q3]	37.5 [21.3, 55.0]	20.0 [10.0, 25.0]	0.06	5.0 [0.0, 12.5]	25.0 [8.8, 50.0]	0.01
Rheumatoid factor (RF)			0.43			0.43
Positive, *n* (%)	8 (66.7)	8 (40.0)		8 (66.7)	8 (40)	
Negative, *n* (%)	3 (25.0)	7 (35.0)		3 (25.0)	7 (35.0)	
not available	1 (8.3)	5 (25.0)		1 (8.3)	5 (25.0)	
Anti-citrullinated protein antibodies (ACPA)			0.44			0.44
Positive, *n* (%)	9 (75.0)	10 (50.0)		9 (75.0)	10 (50.0)	
Negative, *n* (%)	3 (25.0)	8 (40.0)		3 (25.0)	8 (40.0)	
Not available	0 (0)	2 (10.0)		0 (0)	2 (10.0)	
^g^CRP (mg/L), median [Q1, Q3]	5.9 [2.9, 18.3]	2.9 [2.9, 5.1]	0.06	2.9 [2.9, 4.3]	2.9 [2.9, 10.0]	0.77
Treatment use						
^h^bDMARDs (user), *n* (%)	6 (50.0)	9 (45.0)	1	5 (41.7)	8 (40.0)	1
^i^csDMARDs (user), *n* (%)	11 (91.7)	17 (85.0)	1	11 (91.7)	16 (80.0)	0.63
Prednisone (user), *n* (%)	6 (50.0)	9 (45.0)	1	5 (41.7)	9 (45)	1

^a^Upper and lower quartiles; ^b^CDAI, Clinical Disease Activity Index; ^c^HAQ, Health Assessment Questionnaire; ^d^VAS, visual analog scale; ^e^pt_vas, Patient global evaluation of disease activity; ^f^md_vas, Provider global evaluation of disease activity; ^g^CRP, C-reactive protein; ^h^bDMARDs, biologic disease-modifying anti-rheumatic drugs (Abatacept, Adalimumab, Certolizumab, Etanercept, Infliximab, Rituximab, Tocilizumab); ^i^csDMARDs, conventional synthetic disease-modifying anti-rheumatic drugs (Azathioprine, Hydroxychloroquine, Leflunomide, Methotrexate, Sulfasalazine); ^*^Patients were stratified into two groups (MCII+ or MCII−) depending on whether minimum clinically important improvement (MCII) was achieved at follow-up visit; ^**^Fisher’s exact test and Mann-Whitney *U* test was used to test for statistical significance among categorical and continuous variables, respectively

### MCII patient group explains significant variance in gut microbial community composition

We performed a PERMANOVA analysis to evaluate the patient characteristics that contribute to the variance in gut microbial communities of patients with RA (Methods). Using univariate (marginal) models, as well as multivariate (adjusted) models that jointly take into consideration all measurable factors, we considered MCII patient group, age group, sex, smoking status, baseline CDAI, and medication use (for csDMARDs, bDMARDs, and prednisone). Of note, we assume that the resulting percent variance explained by each variable in the adjusted model is statistically independent of other variables.

**Fig. 2:**
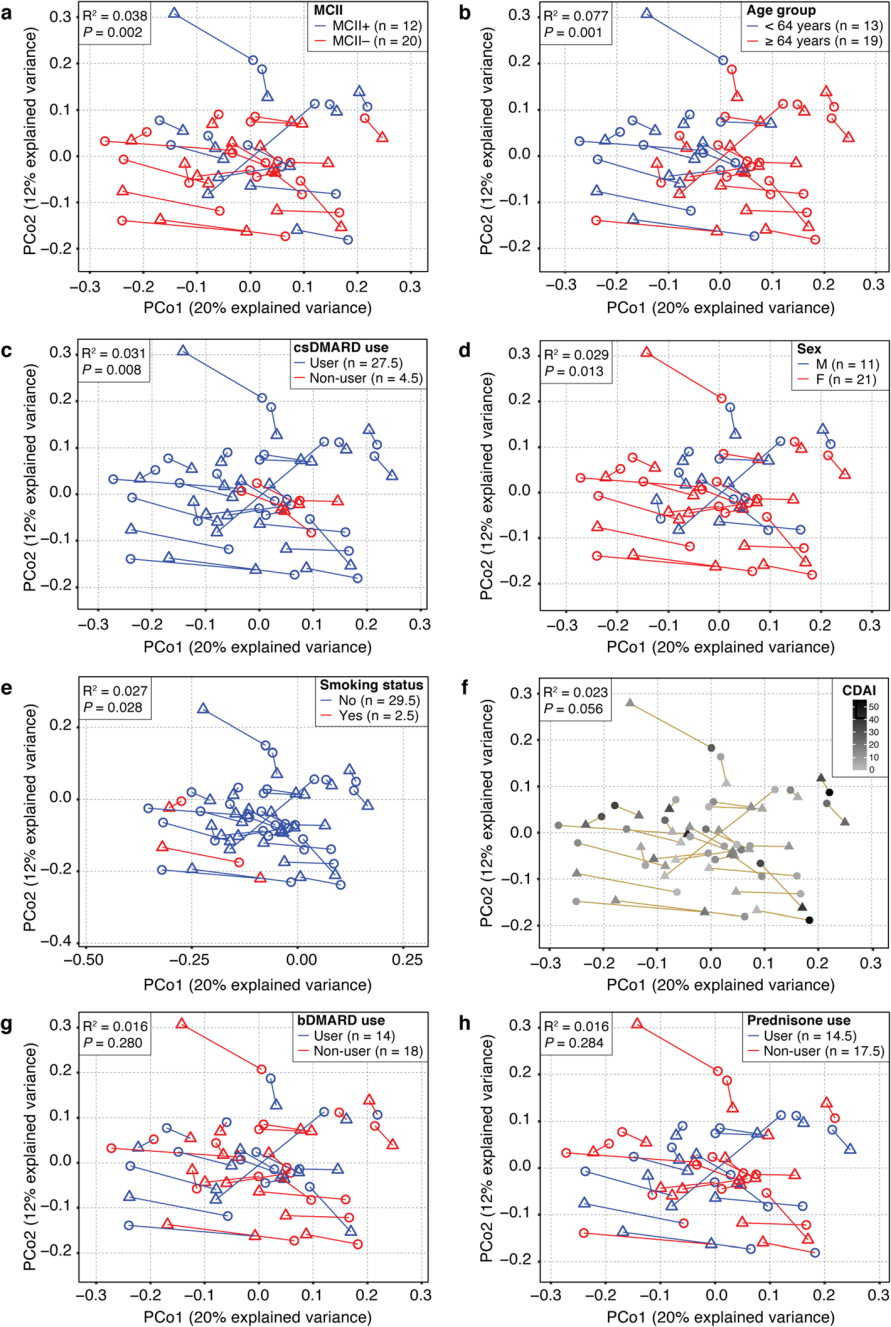
Principal Coordinates Analysis (PCoA) ordination plots of gut microbiome samples from patients with RA (*n* = 32). PERMANOVA analysis finds that the variance in gut microbial community composition can be explained by **a** MCII patient group, **b** age group, **c** use of csDMARDs, **d** sex, **e** smoking status, but not by **f** CDAI, **g** use of bDMARDs, nor **h** use of prednisone. All 64 gut microbiome samples (from 32 patients at both clinical visits) were analyzed simultaneously using PERMANOVA, while intra-subject longitudinal variation was accounted for by constraining permutations to within visits. *R*^2^ and *P* values were derived from the adjusted PERMANOVA models. Each circle and triangle signifies baseline and follow-up, respectively. Lines connect time-points of the same patients. MCII, minimum clinically important improvement. MCII+, patients who showed MCII. MCII−, patients who did not show MCII. Non-integer “*n*” corresponds to cases wherein the patient reported differently at baseline than at follow-up

We found that MCII patient group explained 3.8% of the total variance in gut microbial communities (*P* = 0.002, PERMANOVA; Table 2 and Fig. 2a), after controlling for age group, CDAI, sex, smoking status, use of bDMARDs, csDMARDs, and prednisone, and intra-subject longitudinal variation. The adjusted model also showed that age group, use of csDMARDs, sex, and smoking status significantly explained 7.7%, 3.1%, 2.9%, and 2.7% of the total variance, respectively (Table 2 and Fig. 2b–e), indicating partial dependence of gut microbiome profiles on these other factors; however, CDAI (*P* = 0.056, PERMANOVA; Fig. 2f), treatment with bDMARDs (*P* = 0.280, PERMANOVA; Fig. 2g) and with prednisone (*P* = 0.284, PERMANOVA; Fig. 2h) were not found to have any significant association with gut microbial community composition (Table 2). Taking into account these observations, we additionally controlled for age group, use of csDMARDs, sex, and smoking status in subsequent analyses for investigating the differences in gut microbiome profiles between patients of the MCII+ and MCII− groups.

**Table 2:** Patient characteristics contributing to the variance in gut microbial community composition

Patient characteristics^φ^	Marginal model		Adjusted model	
	Variance (%)	*P* value^*#*^	Variance (%)	*P* value^*#*^
Age group	7.7	0.001	7.7	0.001
MCII patient group	4.4	0.003	3.8	0.002
csDMARDs	3.7	0.008	3.1	0.008
Sex	3.1	0.024	2.9	0.013
Smoking status	4.0	0.009	2.7	0.028
CDAI	2.3	0.120	2.3	0.056
bDMARDs	1.8	0.324	1.6	0.280
Prednisone	1.7	0.350	1.6	0.284

^φ^Each patient characteristic was measured for 32 patients at both clinical visits. All 64 gut microbiome samples were analyzed simultaneously using Permutational Multivariate Analysis of Variance (PERMANOVA); ^#^PERMANOVA was used to test for statistical association between corresponding patient characteristic and variance within microbiome composition

### Features of baseline gut microbiomes significantly differ between MCII+ and MCII− patient groups

At baseline, we observed Bacteroidetes and Firmicutes as the most abundant phyla based upon relative abundances (Additional file 1: Figure S1a); Bacteroidales and Clostridiales as the most abundant orders (Additional file 1: Figure S1b); and Bacteroidaceae as the most abundant family (Additional file 1: Figure S1c). We next investigated the baseline gut microbiomes of all 32 patients to identify differences in ecological diversities (e.g., alpha-/beta-diversity) or in individual taxonomic and functional features between the two MCII patient groups. In effect, by knowing—albeit retrospectively—the clinical outcomes in advance, we have asked: on the basis of gut microbiome information, can differences at baseline not only provide hypotheses that connect gut microbiome to clinical improvement, but also reveal biomarkers predictive of the clinical course?

**Fig. 3:**
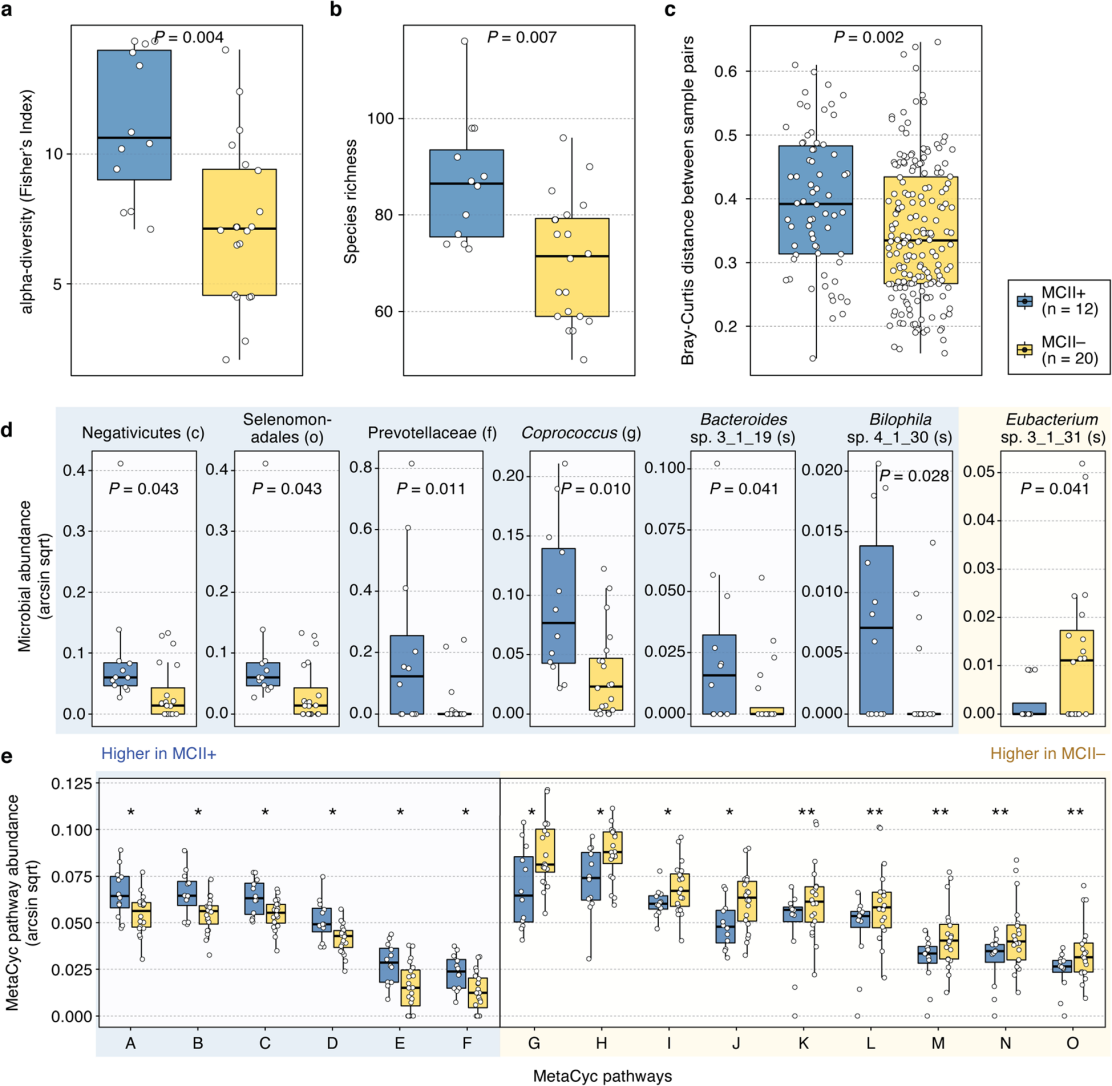
Differences in baseline gut microbiome features between MCII+ and MCII– patient groups. In regard to alpha-diversity, significantly higher species-level **a** Fisher’s Index (*P* = 0.004, MLRM) and **b** richness (*P* = 0.007, MLRM) were observed in the MCII+ group. **c** For beta-diversity, a higher distribution of Bray-Curtis distances between sample-pairs was found in the MCII+ group (*P* = 0.002, Mann-Whitney *U* test). **d** Among the seven microbial taxa found to have significantly different distributions between the two patient groups, six were of higher abundance in the MCII+ group. **e** A total of fifteen MetaCyc biochemical pathways were identified as differentially abundant between the MCII+ and MCII− groups. Except for beta-diversity, multiple linear regression models (MLRMs) were used to test for the statistical significance of the relationship between MCII patient group and microbiome features, while controlling for age group, sex, smoking status, and csDMARD use. *P* value corresponds to the regression model coefficient for the MCII patient group. ^*^, 0.01 ≤ *P* < 0.05; ^**^, 0.005 ≤ *P* < 0.01. MCII, minimum clinically important improvement. MCII+, patients who showed MCII. MCII−, patients who did not show MCII. Taxonomic ranks: c, class; o, order; f, family; g, genus; s, species. MetaCyc pathways: A, Superpathway of S-adenosyl-L-methionine Biosynthesis; B, L-homoserine and L-methionine Biosynthesis; C, Superpathway of L-methionine Biosynthesis; D, L-methionine Biosynthesis I; E, Superpathway of Tetrahydrofolate Biosynthesis and Salvage; F, Superpathway of Tetrahydrofolate Biosynthesis; G, L-ornithine de novo Biosynthesis; H, Superpathway of Pyridoxal 5′-phosphate Biosynthesis and Salvage; I, L-rhamnose Degradation I; J, CMP-3-deoxy-D-manno-octulosonate Biosynthesis I; K, L-arginine Biosynthesis IV; L, L-arginine Biosynthesis I; M, L-arginine Biosynthesis III; N, L-arginine Biosynthesis II; O, L-ornithine Biosynthesis

We found higher species-level alpha-diversity, that is, Fisher’s Index (*P* = 0.004, MLRM; and Fig. 3a) and richness (*P* = 0.007, MLRM; and Fig. 3b), and higher beta-diversity, that is, Bray-Curtis distances between all pairs of samples (*P* = 0.002, Mann-Whitney *U* test; and Fig. 3c) in the MCII+ group compared to the MCII− group. In addition, we sought to identify microbial taxa and microbiome-derived annotated MetaCyc biochemical pathways that were differentially abundant between the two MCII patient groups at baseline. Our analysis uncovered the following six microbial taxa as higher in the MCII+ group: Negativicutes (class), Selenomonadales (order), Prevotellaceae (family), *Coprococcus* (genus), *Bacteroides* sp. 3_1_19 (species), and *Bilophila* sp. 4_1_30 (species), whereas *Eubacterium* sp. 3_1_31 (species) was found to be higher in the MCII− group (*P* < 0.05; and Fig. 3d). Moreover, we found fifteen MetaCyc pathways that were differentially abundant between MCII+ and MCII− groups at baseline (*P* < 0.05, MLRM; and Fig. 3e). Six of these pathways, which include multiple ones for tetrahydrofolate biosynthesis and L-methionine biosynthesis, were significantly more abundant in patients of the MCII+ group than in those of the MCII– group; in contrast, the remaining nine pathways, the majority of which being for L-arginine and L-ornithine biosynthesis, and L-rhamnose degradation, were more abundant in patients of the MCII group. Taken together, our results show that gut microbiomes of the two diverging patient groups start at different ecological states even before reaching their clinical endpoints.

As was in the case at baseline, we observed a significant difference in species-level Fisher’s Index (*P* = 0.037, MLRM; Additional file 1: Figure S2a) between the two MCII patient groups at follow-up visit. However, richness (*P* = 0.094, MLRM) and Bray-Curtis distances between all sample-pairs (*P* = 0.310, Mann-Whitney *U* test) did not show significant differences (Additional file 1: Figure S2b–c). Thirteen microbial clades, including Negativicutes, Bifidobacteriales, and Selenomonadales (order); Bifidobacteriaceae, Prevotellaceae, and Oscillospiraceae (family); *Bifidobacterium* and *Veillonella* (genus); and *Clostridium leptum* and *Roseburia inulinvorans* (species), were found to significantly differ between the MCII+ and MCII− groups at follow-up visit (*P* < 0.05, MLRM; Additional file 1: Figure S2d). Lastly, MetaCyc pathway-level analysis at follow-up visit showed that only “Superpathway of Polyamine Biosynthesis II” was differentially abundant between the two patient groups (*P* < 0.011, MLRM; Additional file 1: Figure S2e).

### Gut microbiome taxa and functions show significant differences in fold-change from baseline to follow-up between MCII patient groups

**Fig. 4:**
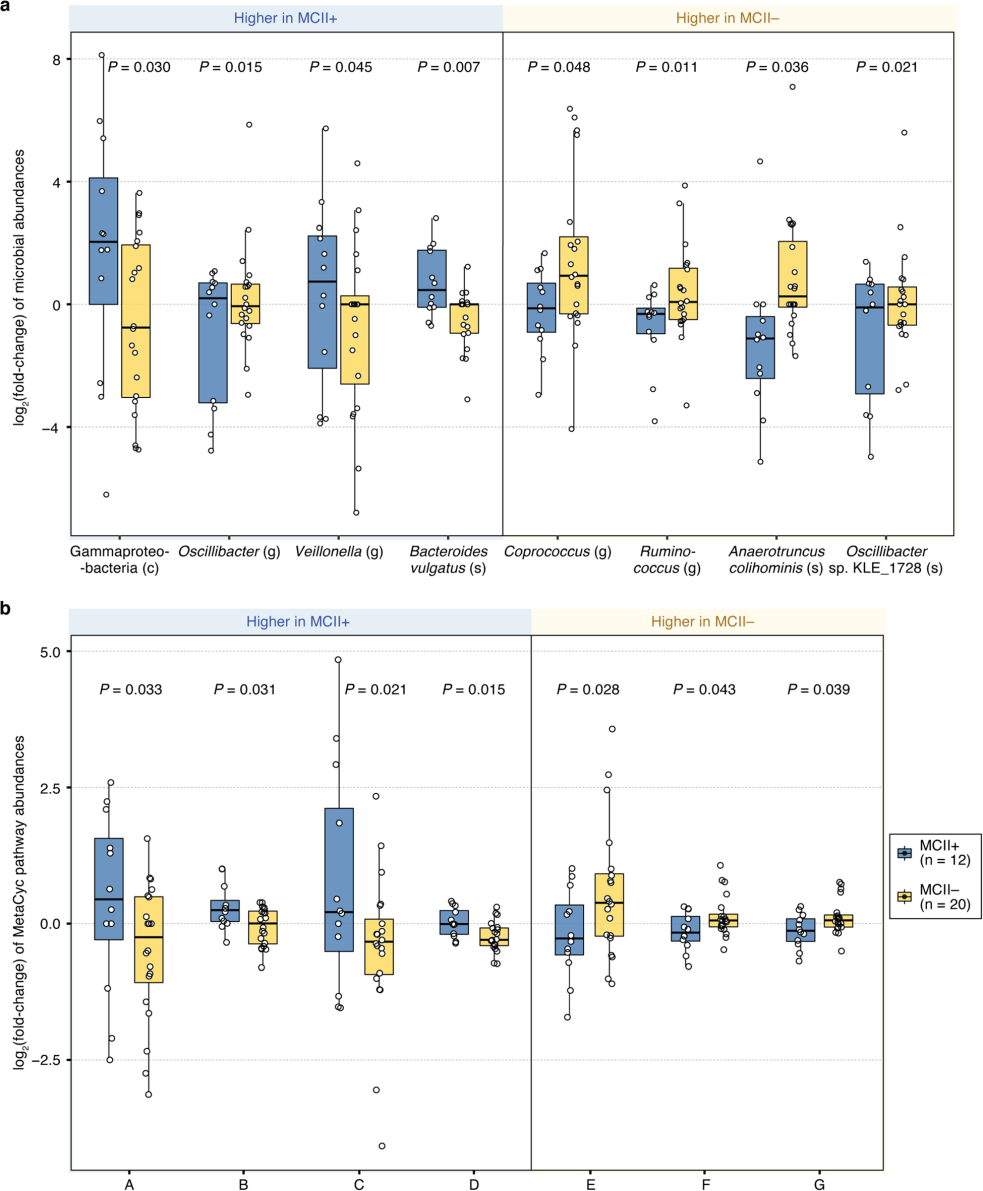
MCII+ and MCII− groups display significantly different fold-changes in microbial taxa and biochemical pathways from baseline to follow-up. **a** Eight microbial taxa showed significant differences in fold-changes (from baseline to follow-up) between the MCII+ and MCII– patient groups (*P* < 0.05, MLRM). Among these eight, relative abundances diverged in opposite directions in five taxa: Gammaproteobacteria and *Oscillibacter* increased in relative abundance from baseline to follow-up in the MCII+ patient group, but decreased in MCII– patient group; alternatively, the relative abundances of *Coprococcus*, *Ruminococcus*, and *Anaerotruncus colihominis* decreased at the follow-up visit in MCII+ patients, but increased in MCII– patients. **b** Seven MetaCyc biochemical pathways were identified as having significantly different fold-changes between the two patient groups (*P* < 0.05, MLRM). Relative abundances diverged in opposite directions in five biochemical pathways: ADP-L-glycero- and beta-D-manno-heptose Biosynthesis (A) and Lipid IVA biosynthesis (C) increased in the MCII+ group, but decreased in the MCII− group; myo-, chiro- and scyllo-inositol Degradation (E), Chorismate Biosynthesis from 3-dehydroquinate (F), and Superpathway of Aromatic Amino Acid Biosynthesis (G) decreased in the MCII+ group, but increased in the MCII− group. *P* values shown above the box plots were found using multiple linear regression models (MLRMs) designed to test for the statistical significance of the association between MCII patient group and fold-change in relative abundances of microbial taxa/pathways. These models were controlled for the following patient factors: age group, sex, smoking status, duration (days) between baseline and follow-up visits, and use of csDMARDs. ^*^, 0.01 ≤ *P* < 0.05. Taxonomic ranks: c, class; o, order; f, family; g, genus; s, species. MetaCyc pathways: A, ADP-L-glycero- and beta-D-manno-heptose Biosynthesis; B, L-rhamnose degradation I; C, Lipid IVA biosynthesis; D, GDP-mannose Biosynthesis; E, myo-, chiro- and scillo-inositol Degradation; F, Chorismate Biosynthesis from 3-dehydroquinate; G, Superpathway of Aromatic Amino Acid Biosynthesis

We examined the longitudinal variation in relative abundances (i.e., fold-change from baseline to follow-up) of microbial taxa and of biochemical pathways. From this, we sought to identify differences in how the gut microbiome changes in association with clinical outcomes (i.e., showing clinical improvement or not). First, we found that patients of the MCII+ and MCII− groups showed significant fold-change differences in the following eight microbial taxa (*P* < 0.05, MLRM; Fig. 4a, Additional file 1: Figure S3a): (i) Gammaproteobacteria (class), *Oscillibacter* (genus), *Veillonella* (genus), and *Bacteroides vulgatus* (species) were higher in the MCII+ group. This result suggests that these four taxa increased in relative abundance more highly and/or frequently in the MCII+ group compared to the MCII− group; and (ii) *Coprococcus* (genus), *Ruminococcus* (genus), *Anaerotruncus colihominis* (species), and *Oscillibacter* sp. KLE_1728 (species) were higher in the MCII− group. In other words, these four taxa increased in relative abundance more highly and/or frequently in the MCII− group than in the MCII+ group.

In the MCII+ group, the relative abundances of four taxa (Gammaproteobacteria, *Oscillibacter*, *Veillonella*, and *Bacteroides*) increased from baseline to follow-up (median log_2_(fold-change) ≥ 0.1), whereas four taxa (*Coprococcus*, *Ruminococcus*, *Anaerotruncus colihominis*, and *Oscillibacter* sp. KLE_1728) decreased in abundance (median log_2_(fold-change) ≤ − 0.1) (Fig. 4a, Additional file 1: Figure S3a). In the MCII− group, the relative abundances of three taxa (*Coprococcus*, *Ruminococcus*, and *Anaerotruncus colihominis*) increased from baseline to follow-up (median log_2_(fold-change) ≥ 0.1), while two taxa (Gammaproteobacteria and *Oscillibacter*) decreased in abundance (median log_2_(fold-change) ≤ − 0.1). Strikingly, these observations imply that the changes in relative abundances (from baseline to follow-up) of Gammaproteobacteria, *Coprococcus*, *Oscillibacter*, *Ruminococcus*, and *Anaerotruncus colihominis* in the MCII+ group and those in the MCII− group generally diverged in opposite directions.

Next, we identified seven biochemical pathways as having significantly different fold-changes between the two MCII patient groups (*P* < 0.05, MLRM; Fig. 4b, Additional file 1: Figure S3b): (i) four pathways, including those involving sugar metabolism (e.g., rhamnose degradation, a heptose derivative biosynthesis, GDP-mannose biosynthesis), had higher fold-changes in the MCII+ group; and (ii) three pathways (“Superpathway of Aromatic Amino Acid Biosynthesis”, “Chorismate Biosynthesis from 3-dehydroquinate”, and “myo-, chiro- and scyllo-inositol Degradation”) had higher fold-changes in the MCII− group.

As seen for microbial taxa, changes in relative abundance of five of these seven biochemical pathways were in opposite directions in the two patient groups: ADP-L-glycero- and beta-D-manno-heptose Biosynthesis, and Lipid IVA biosynthesis (Fig. 4b, Pathway A and C, respectively) generally increased in the MCII+ group, but decreased in the MCII– group; myo-, chiro- and scyllo-inositol Degradation (Fig. 4b, Pathway E), Chorismate Biosynthesis from 3-dehydroquinate (Fig. 4b, Pathway F), and Superpathway of Aromatic Amino Acid Biosynthesis (Fig. 4b, Pathway G) generally decreased in the MCII+ group, but increased in the MCII− group. Although it is yet uncertain why the relative abundances of these particular microbial taxa and biochemical pathways increase (or decrease) in one patient group but decrease (or increase) in the other, such analyses into the changes of distinct gut microbiome features, and how these changes are relevant to clinical improvement, can shed new light on additional insights not provided by cross-sectional datasets.

### Gut microbiome is a predictive marker for clinical improvement and clinical disease activity in patients with RA

**Fig. 5:**
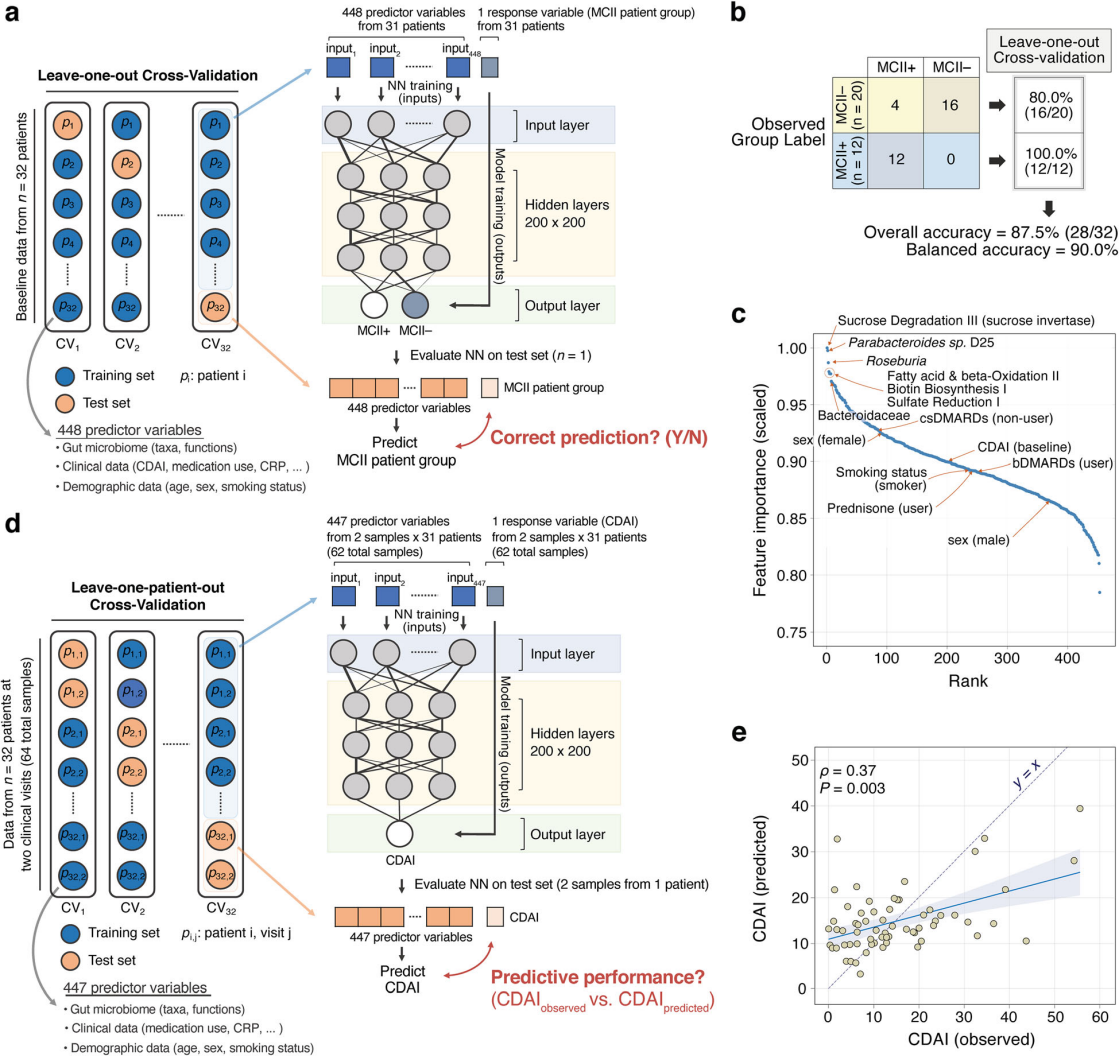
Performance evaluation of neural network-based prediction models in determining minimum clinically important improvement and disease activity score (CDAI). **a** A neural network model was designed to classify patients into one of two MCII patient groups using baseline gut microbiome, clinical, and demographic input features. In leave-one-out cross-validation, this resulted in **b** a confusion matrix of model predictions showing an overall classification accuracy of 87.5% and a balanced accuracy of 90.0%. MCII+, patients who showed MCII. MCII−, patients who did not show MCII. **c** A ranked-order of model input features (total: 448) based upon their scaled (from 0 to 1) importance showing that microbiome data were much more influential contributors to the neural network’s decision-making process than clinical and demographic information. Far left: ranked most important; far right: ranked least important. **d** Another neural network model was constructed to predict CDAI from the same input variables (excluding CDAI) in leave-one-patient-out cross-validation. **e** CDAI predictions were made on both samples from the same left-out patient in each cross-validation loop (see the “Methods” section). In the scatter-plot, predictions made across all 32 iterations of cross-validation are shown simultaneously. Overall correlation between observed and predicted scores: Spearman’s *ρ* = 0.37 (*P* = 0.003; 95% confidence interval: [0.12, 0.58]). Dashed violet line indicates “*y* = *x*,” i.e., an exact match between the observed and predicted values

Having the capability to reliably predict whether a patient will show clinical improvement—independent of prior treatment and clinical course—would address what has been a steep challenge in the clinical practice of RA. As described above, we identified differences in baseline gut microbiome properties between MCII+ and MCII− patient groups. As an extension of these findings, we next turned to the question of how accurately baseline gut microbiome profiles and clinical and demographic data, combined with a machine-learning approach, can predict MCII class for a particular patient or group of patients; this essentially enables us to forecast whether a patient will have a good prognosis, that is, achieving MCII or not. To this end, we used a neural network classification model that incorporates baseline microbiome, clinical, and demographic data as the input variables to classify patients into one of the two MCII patient groups (Fig. 5a; Methods). The neural network model was able to distinguish the two groups with reasonably high prediction accuracy in leave-one-out cross-validation: a balanced accuracy (i.e., average of the proportions of MCII+ and MCII− samples that were correctly classified) of 90.0%, as the classification accuracy for the MCII+ and MCII− group was 100.0% (12 of 12) and 80.0% (16 of 20), respectively (Fig. 5b). Encouragingly, we were able to correctly predict MCII in all twelve patients who did indeed show clinical improvement. Furthermore, the deep-learning neural network provided the best classification performance when compared to logistic regression, support vector machines, and random forests (Methods; Additional file 1: Figure S4), thereby proving its utility over other machine-learning classifiers.

Next, by finding which input features were the most informative in the classification process (Methods), we rank-ordered all features based upon their scaled importance as determined by the neural network. We found that the top-ranked features were mainly composed of taxonomic and functional components from gut microbiome data (Fig. 5c). Of note, the top five important features were the Sucrose Degradation III pathway, *Parabacteroides* sp. D25 (species), *Roseburia* (genus), Fatty Acid and beta-oxidation II pathway, and Biotin Biosynthesis I pathway. Interestingly, data from clinical and demographic characteristics were ranked much lower: the highest ranked non-microbiome feature was related to the use of csDMARDs, which was ranked 78th (out of 448) in regard to feature importance, followed by sex (female), which was ranked 87th. Hence, microbiome features were deemed to be more important to the neural network classifier in predicting the likelihood of clinical improvement.

Surprisingly, the highest ranked (gut microbiome) features of importance were not differentially abundant between the two MCII groups (*P* > 0.05, MLRM; Additional file 1: Figure S5). This seemingly counterintuitive result implies that the most important features to the neural network are not necessarily required to have significant associations with the target variable, that is, MCII status; rather, a nonlinear combination and complex arrangement of the highly ranked features may assert strong predictive power. Alternatively, weakly associated features in unison were actually regarded as important for the supervised classification task.

Having shown that gut microbiome data can be used to predict whether (or not) a patient will show MCII, we developed another neural network model to evaluate how well the aforementioned predictor variables can predict CDAI (Fig. 5d; Methods). The direct prediction of a clinical disease activity score using the gut microbiome has yet to be performed in any chronic disease, although a previous study by Tedjo et al. used a Random Forests classifier with operational taxonomic units (OTUs) of the gut microbiome in Crohn’s Disease to differentiate between active disease and remission [56]. By using a leave-one-patient-out cross-validation scheme, wherein predictions in each cross-validation loop were made on both samples from a single left-out patient (Methods), we found that our neural network achieved a moderate, yet significant, correlation between observed (actual) and predicted CDAI (Spearman’s *ρ* = 0.37, *P* = 0.003; Fig. 5e). Interestingly, the predicted CDAI fits a lower slope compared to the slope of an exact match between observed and predicted values. CDAI beyond ~ 15 were under-predicted, whereas CDAI below ~ 15 were over-predicted; this threshold could possibly indicate a breakpoint at which our model exhibits different relationships between the response and predictor variables. In summary, the gut microbiome shows promise as a non-invasive screening tool for predicting clinical improvement and perhaps also for monitoring RA disease activity.

## Discussion

To the best of our knowledge, this is the first study to date that uses shotgun metagenomic sequencing of stool to investigate the ties between the gut microbiome and MCII in RA disease activity independent of the initial measurement of conditions or prior treatment. This study addresses the following key questions: What are the distinct microbes and functions that define gut ecologies in patients who achieve MCII compared to patients who do not? Are these specific gut microbiome “signatures” predictive of MCII? Or in other words, how well does the gut microbiome forecast the trajectory of RA disease activity irrespective of prior clinical course? To this end, we compared the baseline gut microbiome compositions between RA patients who eventually showed improvement in disease activity and those who did not. First, we found that the status of MCII is significantly associated with the variation in gut microbiome community composition. Next, a more detailed examination of baseline gut microbiomes allowed us to identify higher levels of alpha-diversity (which is often associated with good health) and beta-diversity in the MCII+ group (i.e., patients who achieved clinical improvement) than in the MCII− group (i.e., patients who did not achieve clinical improvement). Additionally, we identified several microbial taxa and microbiome-derived MetaCyc biochemical pathways as differentially abundant between the two MCII patient groups. Furthermore, we observed several taxa and pathways as having significant differences in fold-change (from baseline to follow-up) between the two patient groups. Lastly, we demonstrate that the integration of gut microbiome and machine-learning technology could theoretically be an avenue for the prediction of disease course in RA. More specifically, by incorporating baseline microbiome, clinical, and demographic data into a deep-learning neural network, we were able to effectively classify patients into their MCII+ or MCII group, thereby allowing us to forecast MCII in patients with RA. With further development, such prognostic biomarkers could identify patients who will achieve MCII with a given therapy earlier on, thereby sparing them the expense and risk of other therapies that are less likely to be effective. Conversely, such tools can detect patients whose disease symptoms are less likely to improve, and perhaps allow clinicians to target and monitor them more closely. In all, our proof-of-concept study targets a significant unmet medical need in RA, and demonstrates the utility of the gut microbiome for the precision medicine era.

We identified several microbial taxa at baseline, including *Coprococcus*, *Bilophila* sp. 4_1_30, and Prevotellaceae, to have significantly different relative abundances between the MCII+ and MCII patient groups, even after controlling for demographic and clinical confounders. *Coprococcus* was found to be relatively higher in the MCII+ group compared to the MCII group. Microorganisms of this genus are known to produce butyrate, which is known for its anti-inflammatory effects [57–63]. For example, a study in mice showed that butyrate can suppress inflammation by inhibiting histone deacetylases (HDACs) in bone marrow cells [58]. Previously, the administration of an HDAC inhibitor in vivo was found to promote the production and suppressive function of Foxp3^+^ regulatory T (T_reg_) cells [64]. The anti-inflammatory effect of butyrate was also shown in *Staphylococcus aureus* cell-stimulated human monocytes, to which adding butyrate led to a reduction and increase of proinflammatory cytokine IL-12 and anti-inflammatory cytokine IL-10, respectively [59]. In addition, *Bilophila* sp. 4_1_30 was found to be higher in patients of the MCII+ group. The role of *Bilophila* species in inflammatory or auto-immune diseases is not yet fully understood. A couple of studies have shown the positive association of *Bilophila* species (in particular *B. wadsworthia*) with pro-inflammatory immune responses [65, 66], while another study has shown that *Bilophila* species have negative associations with LPS-induced, TNFɑ-mediated immune responses in whole blood peripheral blood mononuclear cells [67]. Lastly, Prevotellaceae was also found to have greater abundance in the MCII+ group. Some species in this family are known for their pro-inflammatory effects [14, 68]; therefore, this observation possibly suggests that host immune responses to Prevotellaceae are specific to particular species and/or strains [69].

At baseline, 26 of the total 32 patients were on antifolate drugs (methotrexate and/or sulfasalazine). In particular, methotrexate is a folate pathway antagonist known to competitively inhibit dihydrofolate reductase (DHFR), which participates in tetrahydrofolate (THF) biosynthesis [70]. Interestingly, in our study, microbial biochemical pathways involved in tetrahydrofolate biosynthesis at baseline were found to be more abundant in patients of the MCII+ group (Fig. 3e). Although it is yet unclear as to why THF biosynthesis pathways were more abundant in the gut of RA patients who eventually obtained clinical improvement, the elevated presence of these pathways may be possibly linked to a protective role in patient outcome.

In addition to baseline differences in microbial taxa between the MCII+ and MCII− groups, we observed differences in the abundances of fifteen biochemical pathways at baseline. Ten of these differentially abundant pathways are involved in the biosynthesis of amino acids, such as arginine, methionine, and ornithine. All four pathways involved in methionine biosynthesis were found to be more abundant in the MCII+ group. Interestingly, dietary supplementation with high levels of methionine has been shown to attenuate arthritis severity in arthritic rats, and also to increase levels of serum Insulin-like Growth Factor-1 (IGF-I) [71], and to this point, IGF-I was previously found to be significantly lower in female patients with RA than in controls [72]. Alternatively, all four arginine biosynthesis pathways were of lower abundance in the MCII+ group. A recently published study has shown that restriction of arginine improves outcome in multiple murine arthritis models by controlling the metabolism and formation of multi-nuclear giant cells [73].

Patients of the MCII+ and MCII− groups exhibited significantly different fold-changes from baseline to follow-up visit in eight microbial taxa, including *Bacteroides vulgatus*, *Coprococcus*, and *Ruminococcus*, and in seven MetaCyc biochemical pathways, including L-rhamnose degradation I, GDP-mannose Biosynthesis, and Superpathway of Aromatic Amino Acid Biosynthesis (Fig. 4). These differences in fold-changes of microbiome features (taxa/pathways) are likely effects of a complex combination of a number of factors, which could possibly include the use of certain medications. Indeed, several studies have shown that pharmaceutical drugs can be a modulator of gut microbiome composition and metabolic activity [74–76]. In this regard, a recently published study demonstrated that treatment with methotrexate (MTX) in RA patients induced compositional changes in members of the gut microbiota, such as Bacteroidetes, Lachnoclostridium, *Collinsella aerofaciens*, *Dielma fastidiosa*, and *Prevotella copri*, alongside the reduction in multiple immune cell types, which include activated T cells, IFN-γ+ T cells, myeloid cells, and B cells [77]. Along these lines, the use of csDMARDs (which includes methotrexate) was found to be significantly associated with gut microbiome composition in our PERMANOVA analysis. Collectively, our results could implicate various aspects of the gut microbiome with improvement in chronic, debilitating symptoms in RA, raising the interesting possibility of intervening on these markers, e.g., introducing specific desirable bacterial strains into the gut or targeting microbial metabolic pathways as a basis for therapeutic intervention.

Several limitations should be acknowledged when interpreting our results. First and foremost, the relatively small sample size used in our study limits the generalization of the findings to a broader range of RA conditions. It was beyond the scope of this retrospective, observational cohort study to restrict the time of follow-up between clinical visits, leading to variability in the duration of follow-up. While this study is the first to associate gut microbiome signatures with MCII in RA, we do note that our results were derived from a pilot cohort of 32 patients; therefore, conducting more analyses and validation on larger cohorts with pre-specified clinical endpoints is the crucial next step to strengthen and confirm our findings. Second, our results could be influenced by confounders inherent to our cohort of patients. We do acknowledge that there may be geographical/cultural biases in our results, since the patients included in this study are mostly from the midwest region of the United States. Our statistical methods to identify associations between the gut microbiome and MCII were controlled for age, sex, smoking status, follow-up duration, and medication use. However, dietary habits were not assessed, which is a variable well known to influence the composition of the gut microbiome [78, 79]. Importantly, we were not able to statistically control for patient BMI, as current height and weight were found to be missing in several patient records. Of note, obesity is not only strongly tied to gut microbiome [80–82], but also known as a prognostic factor in RA. More specifically, patients with obesity have been found to be less likely to respond to disease-modifying therapy [83]. How much BMI plays a role in shaping the current results will be addressed in our future studies. Third, we lose most of the significant (*P* < 0.05) “hits” found using the MLRMs after Benjamini-Hochberg correction, which could be attributed to a number of factors: (i) lack of strong separation (in gut microbiome) between two study groups having the same disease diagnosis; (ii) comparatively small sample sizes; and (iii) controlling for several potentially confounding factors simultaneously. Fourth, as is often the case in retrospective cohort studies, we cannot completely eliminate the possibility of patient selection bias. For example, patients may not elect to return for a follow-up visit depending on a certain disease severity. Additionally, among the patients whose clinical samples were available in our biobank, some clinical/demographic data were incomplete for both time-points. Such reasons result in exclusion of these patients from our study, and therefore may bias the type of patients who were analyzed. Fifth, all descriptions of annotated biochemical pathways of the gut microbiome allude to *functional potential*, that is, functional possibilities derived from genetic content. We did not employ transcriptomics or proteomics technologies to assess enzyme abundances; metabolomics to detect small-molecules, or cellular assays to determine metabolic flux. However, these are all promising methods that we can later use to obtain much richer insight into how microbial metabolism affects RA disease course. Sixth, clearly our study cannot provide causal mechanisms underlying the associations between the gut microbiome and MCII in RA disease activity. However, a closer investigation on particular microbial taxa or microbiome-derived pathways identified in our study may provide a promising launchpad for future studies delving into specifically how alterations in the gut microbiome influence RA-associated changes in human physiology or in systemic, chronic inflammation. Seventh, all predictions regarding the MCII patient group and CDAI were performed in cross-validation on the original discovery cohort. It remains to be seen how well the robustness of our prediction models will hold up when demonstrated on an independent validation cohort once available. Finally, although we found that the gut microbiome is surprisingly predictive of MCII, our study is limited by the fact that we collected stool samples and assessed patients’ disease activity at only two time-points. It could be possible that associations between gut microbiome and MCII may not persist past the second visit. Surely, future studies extending this current work will need to entail having larger cohorts, patients with new-onset RA, and several longitudinal sample collections, while considering more potentially confounding factors (e.g., geography, race/ethnicity, diet, and lifestyle).

## Conclusions

Several aspects of the gut microbiome are associated with future prognosis in RA, providing motivation for further studies on the effect of intestinal microflora and various patient factors on autoimmune response and clinical course. Additionally, shotgun metagenomic sequencing of microbial communities in stool samples can serve as an effective and reliable predictor of whether patients with RA will achieve clinically important improvement in disease activity. Ultimately, we expect our work to be one cornerstone for a suite of new, omics data-based clinical tools to aid in early detection, diagnosis, prognosis, and treatment in RA [84, 85]. Looking ahead, possible solutions to treat chronic auto-immune or inflammatory diseases could well involve modifying the gut microbiome to an ecological state primed to enhance clinical outcome.

## Supplementary Information

**Additional file 1: Figure S1.** Stacked bar-plots showing the distribution of relative abundances of taxonomic ranks detected in baseline gut microbiomes. **Figure S2.** Differences in gut microbiome features between MCII patient groups at follow-up visit. **Figure S3.** Microbial taxa and biochemical pathways whose change in relative abundance from baseline to follow-up vary differently between MCII patient groups. **Figure S4.** Performance evaluation of three different classifiers to predict MCII status. **Figure S5.** Relative abundances of the top 10 highest-ranked features in the deep-learning neural network model.

## Data Availability

Sequencing data for stool metagenomes used in this study have been deposited at NCBI’s Sequence Read Archive (SRA) data repository (BioProject number PRJNA598446 [86] and PRJNA687957 [87]) and can be downloaded without any restrictions. The deposited sequences include .fastq files for 64 stool metagenomes collected from 32 patients with rheumatoid arthritis. Human reads were identified and removed prior to data upload.

## Abbreviations

bDMARDs: Biologic disease-modifying anti-rheumatic drugs
CDAI: Clinical disease activity index
CRP: C-reactive protein
csDMARDs: Conventional synthetic disease-modifying anti-rheumatic drugs
DAS: Disease Activity Score
HAQ: Health Assessment Questionnaire Disability Index
IL: Interleukin
MCII: Minimum clinically important improvement
PERMANOVA: Permutational multivariate analysis of variance
RA: Rheumatoid arthritis
SDAI: Simplified disease activity index
SJC: Swollen joint count
TJC: Tender joint count
