# Supplementary material for: Gut microbial determinants of clinically important improvement in patients with rheumatoid arthritis
Source: Genome Med. 2021 Sep 14;13:149. doi: 10.1186/s13073-021-00957-0 (PMC8439035; doi:10.1186/s13073-021-00957-0)
Supplement: Supplementary file 1 — Additional file 1: Figure S1. Stacked bar-plots showing the distribution of relative abundances of taxonomic ranks detected in baseline gut microbiomes. Figure S2. Differences in gut microbiome features between MCII patient groups at follow-up visit. Figure S3. Microbial taxa and biochemical pathways whose change in relative abundance from baseline to follow-up vary differently between MCII patient groups. Figure S4. Performance evaluation of three different classifiers to predict MCII status. Figure S5. Relative abundances of the top 10 highest-ranked features in the deep-learning neural network model. [file 13073_2021_957_MOESM1_ESM.pdf]

Gut Microbial Determinants of Clinically Important Improvement in Patients with Rheumatoid Arthritis

Gupta *et al.*

Additional file 1: Figures S1–S5

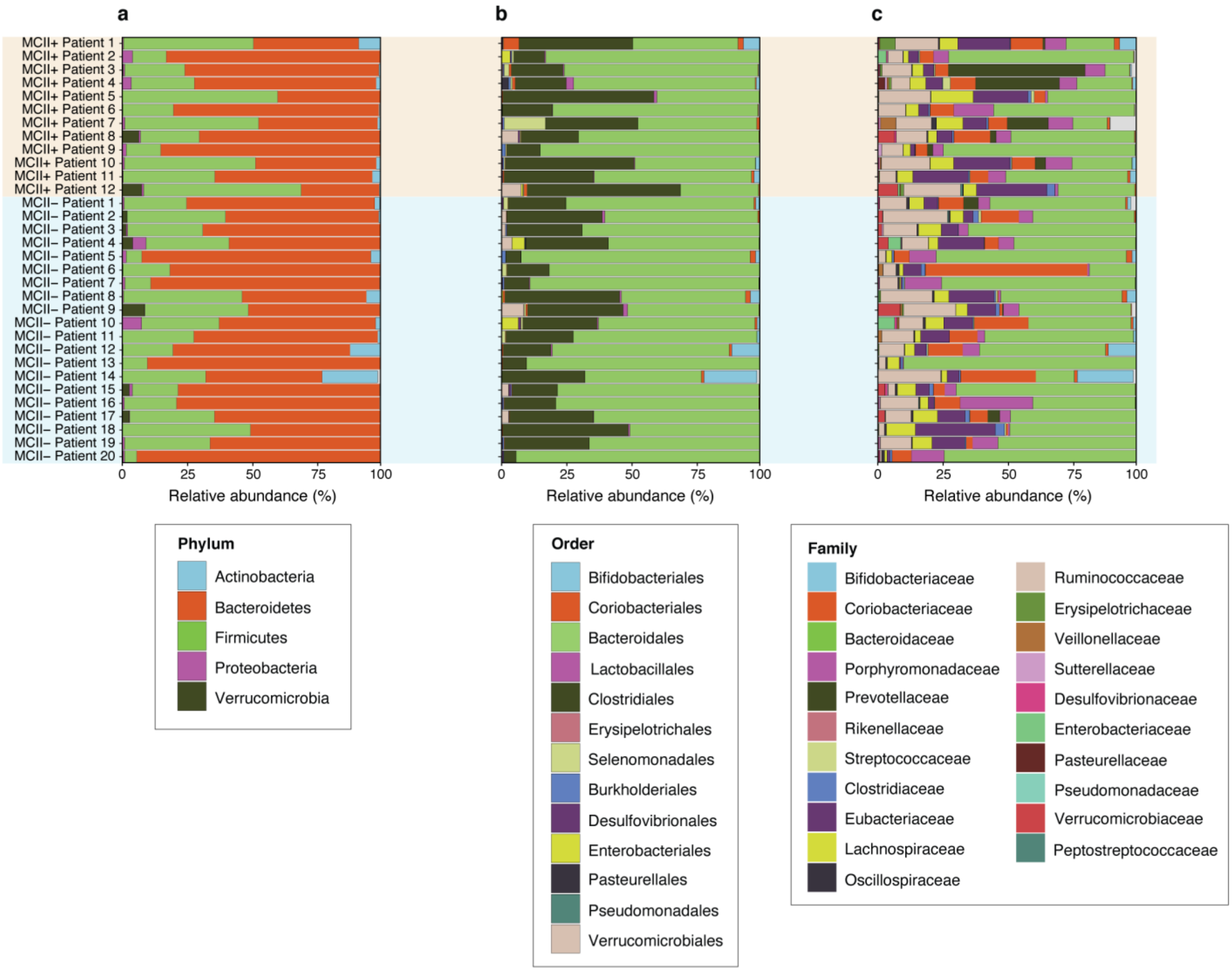

Figure S1. Stacked bar-plots showing the distribution of relative abundances of taxonomic ranks detected in baseline gut microbiomes. At **(a)** phylum-level, Bacteroidetes and Firmicutes were the two most abundant. At **(b)** order-level, Bacteroidales and Clostridiales were most abundant. Among **(c)** families, Bacteroidaceae was the most abundant.

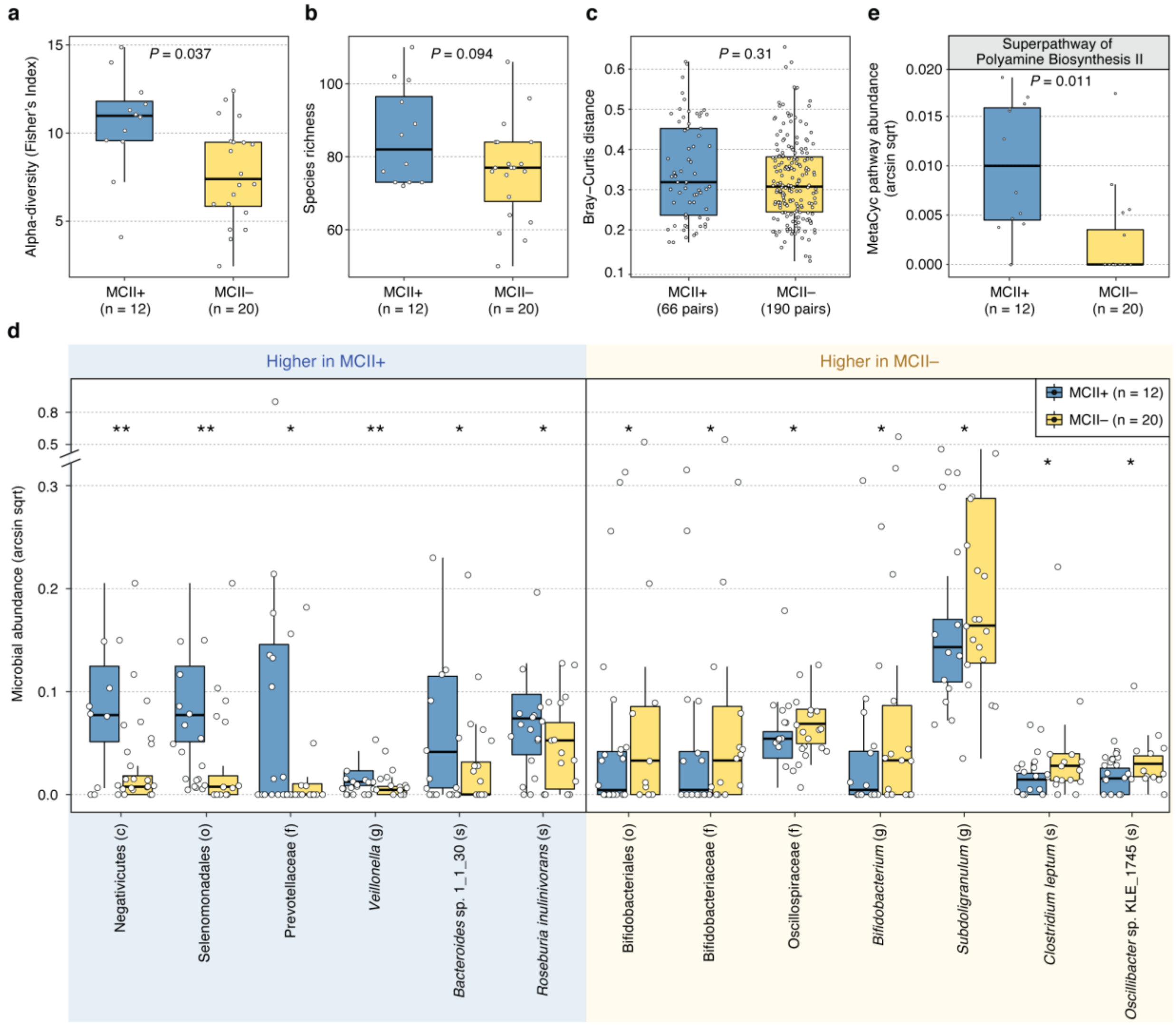

Figure S2. Differences in gut microbiome features between MCII patient groups at follow-up visit. In the gut microbiomes of patients with RA at follow-up, species-level **(a)** Fisher's Index (*P* = 0.037, MLRM) was found to be significantly different between the MCII+ and MCII- groups, but **(b)** richness (*P* = 0.094, MLRM), and **(c)** Bray-Curtis distances between all sample-pairs (*P* = 0.31, Mann-Whitney *U* test) were not found to be significantly different. **(d)** Thirteen microbial taxa and **(e)** one MetaCyc biochemical pathway were identified as differentially abundant between the two MCII groups. All MLRMs were adjusted for patient factors (age group, sex, smoking status, and use of csDMARDs) reported at follow-up. Microbial taxa and biochemical pathways were considered as differentially abundant if the regression model coefficient for MCII patient group was significant ($P < 0.05$). MCII, minimum clinically important improvement. MCII+, patients who showed MCII. MCII-, patients who did not show MCII. *, $0.01 \leq P < 0.05$; **, $P < 0.01$. Taxonomic ranks: c, class; o, order; f, family; g, genus; s, species.

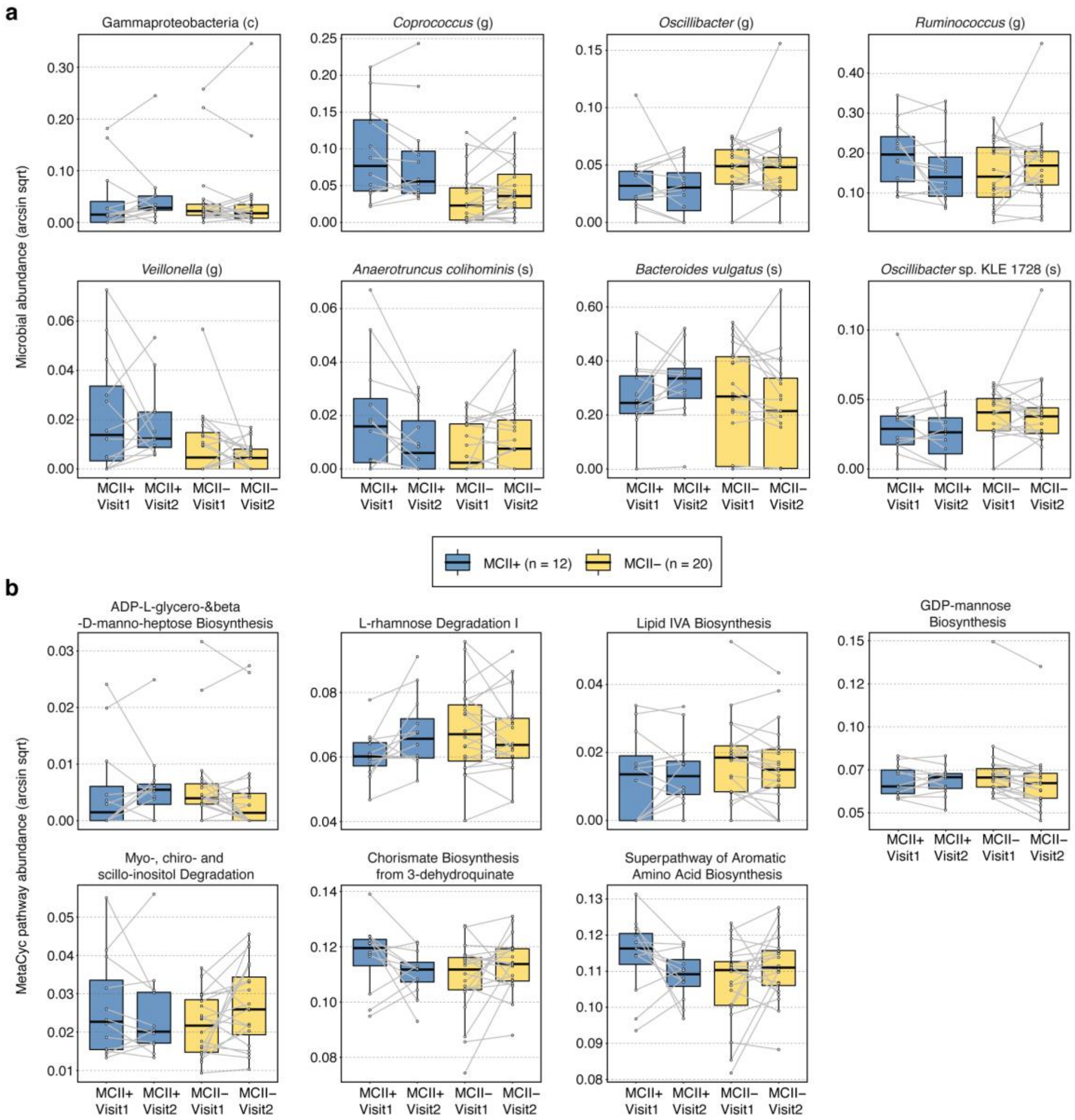

Figure S3. Microbial taxa and biochemical pathways whose change in relative abundance from baseline to follow-up vary differently between MCII patient groups. (a) Eight microbial taxa (Gammaproteobacteria, *Coprococcus*, *Oscillibacter, Ruminococcus, Veillonella, Anaerotruncus colihominis, Bacteroides vulgatus*, and *Oscillibacter* sp. KLE 1728) displayed significantly different fold-changes between the two MCII patient groups. **(b)** Seven MetaCyc biochemical pathways (ADP-L-glycero- & beta-D-manno-heptose Biosynthesis, L-rhamnose degradation I, Lipid IVA biosynthesis, GDP-mannose Biosynthesis, myo-, chiro- and scillo-inositol Degradation, Chorismate Biosynthesis from 3-dehydroquinate, and Superpathway of Aromatic Amino Acid Biosynthesis)

showed significantly different fold-changes between the two groups. Points connected by gray lines indicate stool metagenome (gut microbiome) samples from the same patient at two clinical visits. Visit 1: baseline; Visit 2: follow-up. Taxonomic ranks: c, class; o, order; f, family; g, genus; s, species.

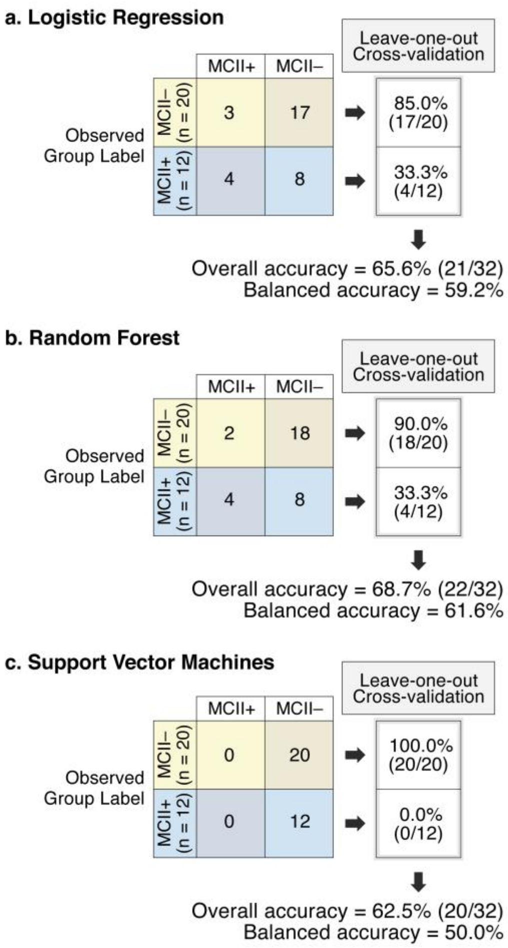

Figure S4. Performance evaluation of three different classifiers to predict MCII status. Baseline gut microbiome data composed of 32 samples and 448 features were used as the training data. Features of the structured matrix were relative abundances of 176 taxonomic ranks, 262 MetaCyc pathways, seven clinical and three demographic information. The predictive performance of each classification model was evaluated by leave-one-out cross-validation. More specifically, in each cross-validation loop, each classifier used 31 samples and 448 features to train the model and used the remaining sample to predict MCII. **(a)** The overall and balanced accuracy of the logistic regression classifier resulted in 65.6% and 59.2%, respectively. **(b)** The overall and balanced accuracy of the random forest classifier resulted in 68.7% and 61.6%, respectively. **(c)** The overall and balanced accuracy of the support-vector machines classifier resulted in 62.5% and 50.0%, respectively. Logistic regression, random forest, and support-vector machine models were available from the 'scikit-learn' package v0.24.1. MCII, Minimum clinically important improvement; MCII+, patients who showed MCII; MCII-, patients who did not show MCII.

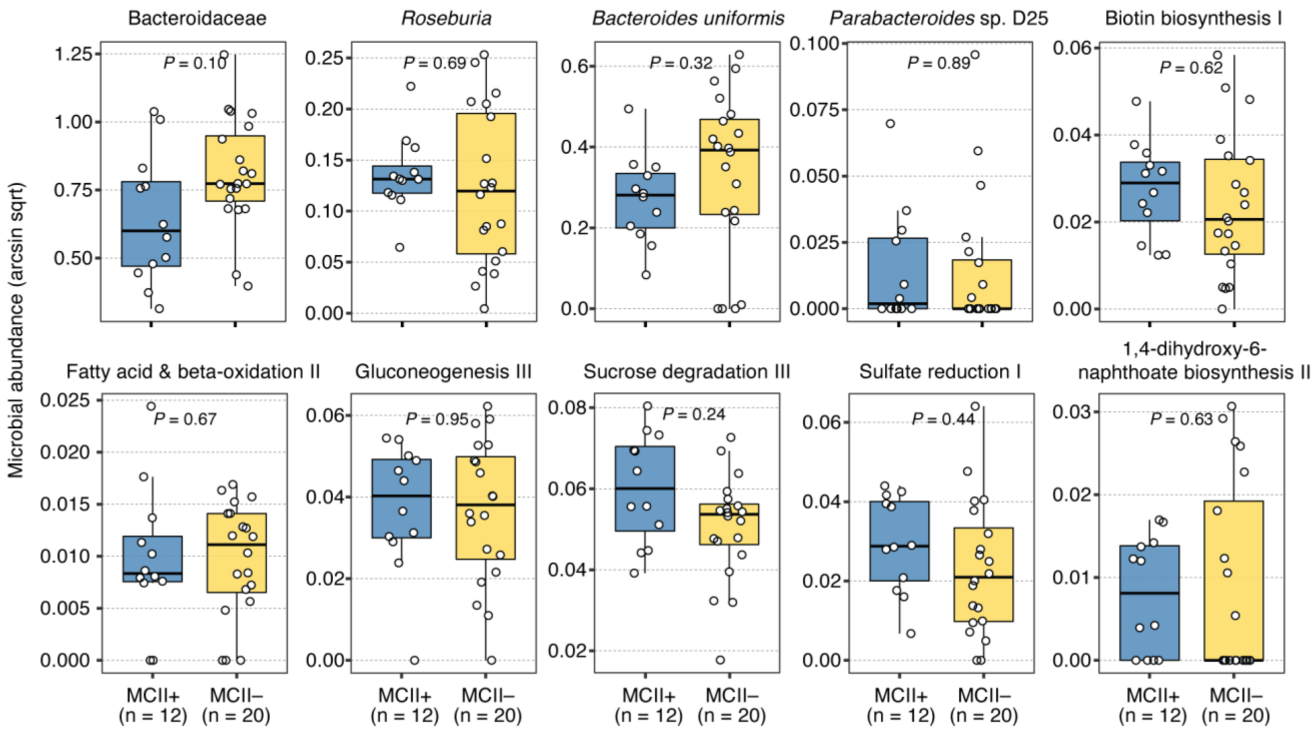

Figure S5. Relative abundances of the top 10 highest-ranked gut microbiome features in the deep-learning neural network model. Multiple linear regression models (MLRMs) were designed to test for the statistical significance of the relationship between MCII patient group and each microbiome feature, while controlling for clinical/demographic factors (age, sex, smoking status, and use of csDMARDs) reported at baseline. *P*-value corresponds to the regression model coefficient for the MCII patient group.
